# The Association of Inflammatory Markers, IL-1*α* and TGF-*β*, with Dietary Insulin Load and Dietary Insulin Index in Overweight and Obese Women with Healthy and Unhealthy Metabolic Phenotypes: A Cross-Sectional Study

**DOI:** 10.1155/2022/3407320

**Published:** 2022-10-13

**Authors:** Sahar Noori, Atieh Mirzababaei, Farideh Shiraseb, Reza Bagheri, Cain C. T. Clark, Alexei Wong, Katsuhiko Suzuki, Khadijeh Mirzaei

**Affiliations:** ^1^Department of Nutrition, Science and Research Branch, Islamic Azad University, Tehran, Iran; ^2^Department of Community Nutrition, School of Nutritional Sciences and Dietetics, Tehran University of Medical Sciences (TUMS), Tehran, Iran; ^3^Department of Exercise Physiology, University of Isfahan, Isfahan, Iran; ^4^Centre for Intelligent Healthcare, Coventry University, Coventry CV1 5FB, UK; ^5^Department of Health and Human Performance, Marymount University, Arlington, USA; ^6^Faculty of Sport Sciences, Waseda University, 2-579-15 Mikajima, Tokorozawa, Saitama 359-1192, Japan

## Abstract

*Context* Research has shown IL-1*α* might play a role in the associations between the MH group and DII and DIL. *Objective*. We evaluated the association of inflammatory markers, IL-1*α* and TGF-*β*, with dietary insulin load and index in women with healthy and unhealthy obesity phenotypes. *Materials and Methods*. 228 obese/overweight women aged 18–48 years were included in this study. Biochemical factors were obtained from blood samples. Body composition, anthropometric measures, and physical activity assessments were performed. Dietary intakes, DII, and DIL were assessed. *Results*. Significant associations were observed between the MH group and the DII group (OR = 2.142, 95% CI = 1.421, 2.850, and *p* = 0.040), in which IL-1*α* may play a role. *Discussion and Conclusion*. Significant associations were observed between the MH group and DII. IL-1*α* might play a role in these associations.

## 1. Introduction

Obesity is a public health problem whose global prevalence has dramatically increased in recent decades[1–3]. According to the World Health Organization (WHO), more than 1.9 billion adults worldwide are overweight, while about a third of the world's population is obese [4]. Several studies have shown that obesity-induced inflammation can cause and contribute to comorbidities and complications, such as metabolic syndrome (MetS), insulin resistance (IR), cardiovascular disease, nonalcoholic fatty liver disease, and certain cancers [5–7]. Therefore, inflammatory molecules may be an interesting therapeutic target to prevent and manage the comorbidities of obesity.

Visceral adipose tissue is an endocrine-like tissue that produces and secretes various substances, such as adipokines, and performs various biologic functions, such as regulating metabolic processes, insulin secretion, hunger and satiety, energy balance, and inflammation [8]. Interleukin-1*α* (IL-1*α*), tumor necrosis factor-*α* (TNF-*α*), leptin, and transforming growth factor-*α* (TGF-*α*) are some of the main adipokines involved in the regulation of inflammatory processes [8] in obese individuals [9]. Increasing the secretion and function of IL-1*α*, locally and systemically, causes IR [10–18]. Additionally, elevations in TGF-*α* have been associated with obesity and obesity-related comorbidities such as diabetes and dyslipidemia [19–21], indicating the potential role of adipokines in the metabolic profile of obese cohorts.

Recently, metabolic phenotypes have been used to identify new features of obesity. Metabolically healthy obesity (MHO) is a healthy obesity phenotype found in young obese individuals who are physically active, have better nutritional status and lower visceral and ectopic fat storage [9], with a body mass index (BMI) above 30 kg/m^2^, and have no risk factors for cardiometabolic diseases compared to normal-weight individuals [22]. Indeed, studies have shown that individuals with MHO have higher physical activity levels than those with metabolically unhealthy obesity (MUO), which indicates that physical activity and lifestyle may cause a healthier metabolic profile in obese cohorts [23]. Moreover, individuals with MHO have no concomitant metabolic abnormalities, such as IR, high blood pressure (BP), and dyslipidemia, and tend to have lower concentrations of systemic inflammation [24]. However, metabolically unhealthy obesity (MUO) is in contrast to MHO. Indeed, changes in the function and distribution of adipose tissue underlie the MUO phenotype where lower subcutaneous fat mass, preinflammatory, and impaired fat storage capacity in adipose tissue may lead to more visceral fat accumulation in liver tissue and skeletal muscle. Inflammation in visceral adipose tissue helps develop insulin resistance and chronic cardiovascular disease [25]. It has been previously established that some modifiable lifestyle factors, such as dietary habits, can affect metabolic phenotypes, IR, and chronic inflammation [22, 26, 27]. Recent studies suggest that high levels of dietary acid load can affect glucose metabolism and insulin sensitivity [28]. Consequently, evaluating different dietary indices may be clinically relevant to implementing successful prevention and treatment strategies in overweight and obese populations.

The dietary insulin index (DII) expresses insulin responses to isoenergetic components of foods compared to the reference food, which is based on postprandial insulin secretion [29]. This index considers the carbohydrate content of foods and high-protein and high-fat compounds and their interactions [30]. Dietary insulin load (DIL) is another index calculated by multiplying the values of DII of each food by the energy content and frequency of consumption of each food [31]. Recent studies have shown that diets with a high insulin index are associated with increased odds of obesity in women [32–34]. Indeed, such diets can cause or contribute to excessive insulin secretion, increased oxidative stress, beta-cell dysfunction, and an increased risk of obesity and type 2 diabetes [34]. It has been previously reported that elderly men with high DIL had elevated C-reactive protein (CRP) concentrations compared to those with low DIL [35].

On the other hand, Nimptsch et al. found no associations between the DII or DIL and inflammatory markers such as CRP and IL-6 in healthy individuals [36]. However, no study has examined the associations between DII or DIL and inflammatory factors in cohorts with MHO and MUO. Therefore, this study aimed to investigate the relationship of inflammatory factors (IL-1*α* and TGF-*α*) with DII and DIL on healthy and unhealthy metabolic phenotypes in adult obese and overweight women. We hypothesized that DII and DIL could affect fat accumulation, fat distribution, inflammatory factors, and the metabolic phenotypes of obesity via different insulin responses.

## 2. Materials and Methods

### 2.1. Participants

Two hundred and twenty eight obese/overweight women from Tehran, Iran, were recruited as potential participants and screened based on the following inclusion/exclusion criteria. Inclusion criteria included (1) age 18–48 years, (2) lack of additional/unusual physical activity, no smoking, no alcohol consumption or usage of supplements or weight loss drugs, (3) no history of type 2 diabetes, cardiovascular disease, polycystic ovary syndrome, stroke, nonalcoholic fatty liver disease, inflammatory diseases, hypertension, cancer, and thyroid disease. Exclusion criteria included (1) pregnancy, breastfeeding, eating disorders, or having chronic diseases; (2) individuals who did not answer more than 70 questions on the semiquantitative food frequency questionnaire (FFQ), or whose daily energy intake was outside the range of 800–4200 kcal, as well as those taking drugs that could create weight changes or affect BP, blood lipoproteins, and blood glucose were excluded from the study. With the following specifications, the study protocol was approved by the Tehran University of Medical Sciences (TUMS) Ethics Committee with the following identification: IR.TUMS.VCR.REC.1398.463.

### 2.2. Study Design

In this cross-sectional study, sampling and data collection were performed in 2017–2018. Random cluster sampling was performed among health centers affiliated with the Tehran University of Medical Sciences (TUMS). Two visits were conducted: in the first visit, a demographic questionnaire, food frequency questionnaire, and anthropometric and body composition measurements were performed. On the second visit, blood samples were taken from individuals.

### 2.3. Assessment of Biochemical Factors and Inflammatory Markers

Blood samples were collected and stored in tubes containing 0.1% EDTA, after 10–12 hours of fasting, according to the standard protocol at the Nutrition and Biochemistry laboratory of the School of Nutritional Sciences and Dietetics, TUMS. To collect serum, the samples were centrifuged at 3000 rpm for 10 minutes, divided into 1 ml tubes and stored at −70°C until analysis by an autoanalyzer BT 1500 (Selectra 2; Vital Scientific, Spankeren, Netherlands).

Fasting blood sugar (FBS) was measured by glucose oxidase phenol 4-aminoantipyrine peroxidase (GOD/PAP) method on the day of sample collection. Blood triglyceride (TG) concentration was measured with triacylglycerol kits (Pars Azmoon Inc., Tehran, Iran) using enzymatic colorimetric tests with glycerol-3-phosphate oxidase phenol 4-aminoantipyrine peroxidase (GPO-PAP). Total-cholesterol (total-chol) concentration was assayed by the cholesterol oxidase phenol 4-aminoantipyrine peroxidase (CHOD-PAP); low-density lipoprotein (LDL) and high-density lipoprotein (HDL) were measured by the direct method and immunoinhibition. Serum insulin concentrations were analyzed through the enzyme-linked immunosorbent assay (ELISA) method (human insulin ELISA kit, DRG Pharmaceuticals, GmbH, USA), and the minimum detectable concentration was 1.76 mlU/ml. All kits were from Pars Azmoon (Pars Azmoon Inc., Tehran, Iran). Circulating inflammatory markers were measured using immunoassay methods.

### 2.4. The HOMA-IR Calculation

IR was measured by the homeostatic model assessment (HOMA): HOMA-IR = [fasting plasma glucose (mmol/l) × fasting plasma insulin (mIU/l)]/22.5 (36).

### 2.5. Assessment of Anthropometric and Body Composition Indices

Anthropometrics and body composition measurements occurred between 8 and 9 am after 12 hours of overnight fasting. Participants were asked to avoid any unusual physical activity for 72 h before the assessment. Height was determined to the nearest 0.1 cm with a stadiometer, while body mass was measured with InBody 770 Scanner (Inbody Co., Seoul, Korea). BMI was calculated by dividing the body mass (kg) by the height (cm) square. Waist circumference (WC), at the smallest girth, and hip circumference (HC), at the largest girth, were measured with an accuracy of 0.1, according to standard anthropometric guidelines [37].

Body composition was assessed by using a multifrequency bioelectrical impedance analyzer device (BIA): InBody 770 Scanner (Inbody Co., Seoul, Korea). This device measures the resistance of body tissues by sending electrical signals sent from both hands and feet. 30 min before the test, participants were asked to urinate (void) completely and avoid consuming water. According to the manufacturer's instructions, participants were asked to remove all metal tools, jewelry, coats, jackets, and shoes [36]. To increase the device's accuracy, they were also asked to take off their socks before being placed on them. Participants stood on the balance scale in bare feet and held the handles of the device; the amount and proportion of body fat percentage (BF%), fat-free mass (FFM), fat mass (FM), fat-free mass index (FFMI), fat mass index (FMI), trunk fat, waist-to-height ratio (WHR), and waist-to-height ratio (WHtR) were then measured. The retest reliability of our BIA in our lab is *r* = 0.98.

### 2.6. Assessment of Blood Pressure

After 15 minutes of rest, BP was measured by a trained physician with a standardized sphygmomanometer (Omron, Germany, European). Two measurements at 1-minute intervals were collected and averaged.

### 2.7. Metabolic Health and Its Components

We used Karelis' criteria to define metabolic health, assessing inflammatory profiles and insulin sensitization to other indicators. According to Karelis criteria, the presence of 4 or more of the following items indicates a healthy phenotype: TG ≤ 1.7 mmol/l, HDL≥1.3 mmol/l, and no treatment, LDL ≤2.6 mmol/l, hs-CRP≤ 3.0 mg/l, and HOMA-IR ≤2.7 (38). The presence of 3 or fewer items was considered an unhealthy phenotype (39–41).

### 2.8. Assessment of Dietary Intake

Food intake was assessed by using the 147-item semiquantitative FFQ questionnaire. Frequent intake for each food was converted to daily intakes, and then the portion of each food was converted to grams by using Nutritionist 4 (N4). The FFQ questionnaire used in this article has been validated and used in previous literature [38–41].

### 2.9. Calculation of DII and DIL

The insulin index of each food was extracted for analysis, based on the methods outlined in [42]. DII is the area under the curve, indicating the insulin response induced by food intake with a content of 1000 kJ of energy within 2 hours, which is divided by the area under the curve due to the reference consumption of isogenic food [29].

The following formula was used to calculate the dietary insulin load [34]:

(1) $$\begin{array}{c} insulin\;loa\;d\;of\;foo\;d=\sum \left[insulin\;in\;de\;x\;of\;foo\;d\times energy\;content\;of\;foo\;d\left(kcal/serving\right)\times frequency\;of\;consumption\left(serving\;of\;foo\;d/da\;y\right)\right]. \end{array}$$

### 2.10. Physical Activity

Participants' oral responses to International Physical Activity Questionnaire (IPAQ) were collected through interviews and reported as metabolic equivalent h/wk (34) (MET-h/wk). This questionnaire included five sections of questions in physical activity: (1) work-related physical activity, (2) housework, (3) transportation, (4) physical activity during leisure -time and sports, and (5) the time spent sitting. Participants responded according to the intensity (moderate or severe) and the length of time they were engaged in these activities during the last seven days. Physical activity was classified as follows: low <600 (MET-h/wk), moderate = 600–3500 (MET-h/wk), and severe >3500 (MET-h/wk) [43].

### 2.11. Statistical Analysis

All statistical analyses were performed using statistical package for social sciences (version 25.0; SPSS Inc, Chicago, IL, USA). A total sample size of 228 participants was determined with the following formula: *n*=(([(*Z*_1−*α*_+*Z*_1−*β*_) × √1−*r*^2^]/*r*)^2^+2), where *β* = 0.95, *r* = 0.25, and *α* = 0.05 [44]. The data had a normal distribution, which was confirmed using the Kolmogorov Smirnov test. *P* values <0.05 were, a priori, considered statistically significant. Quantitative variables were reported with a mean (SD), and categorical variables were reported by number and percentage. Statistical analyses for quantitative variables were performed using an independent sample *t*-test between two categories of metabolically healthy (MH) and metabolically unhealthy (MUH), and analysis of covariance (ANCOVA) was used, adjusted for confounders of age, physical activity, BMI, and energy intake (kcal). Pearson correlation analysis was utilized to assess the relationship between two inflammatory factors with DII and DIL; correlation coefficients (*r*) and *P* values were reported in this analysis. The binary logistic regression method was used to examine the association of TGF-*α* and IL-1*α* and MH and MUO and DII and DIL with MH and MUO in overweight and obese women. Odds ratios (ORs) and 95% CI were reported for this analysis. In this study, the reference group was metabolically healthy, and the confounding factors were age, physical activity, energy intake, BMI, TGF-*α*, IL-1*α*, FM, and FFM.

## 3. Results

Two hundred and twenty eight participants were included in the present analysis. Our participants had a mean (SD) age of 36.166 (8.332) years and a height of 161.368 (.773) (cm). Moreover, weight was 80.228 (SD = 12.145) (kg) and BMI 30.769 (SD = 4.239) kg.m^2^. For DII and DIL, mean (SD) values were 39.787 (9.863) and 105070.521 (43424.758); while TGF-*α* and IL-1*α* values were 79.476 (SD = 49.457) ng/ml and 2.783 (SD = 0.934) ng/ml, respectively. 29.5% of individuals were MH, while 70.5% were MUH. Among the study population, 26.1% had a high and 24.8% had low economic status, 76.9% were married, and 49.2% were educated to a bachelor's degree or higher.

### 3.1. Characteristics of MH and MUH Phenotypes

The characteristics of obese and overweight women in two categories of MH and MUH are presented in Table 1. Variables including body mass (0.001), BMI (<0.001), FMI (<0.001), BF (%) (0.032), BFM (<0.001), trunk fat (<0.001), WC (0.008), WHR (0.002), and WHtR (<0.001) in the MH and MUH categories were significantly different in both crude model and after adjustment with potential confounders (age, physical activity, energy intake, and BMI). Among these confounders, age was higher in the MH group, while BMI, physical activity, and energy intake were higher in the MUH group. HC was significant in the crude model, and after adjustment with confounders, significance disappeared (*p* > 0.05). HDL and HOMA-IR were significant in the crude model and after adjustment. Regarding other variables and after controlling for confounders, they were not significantly different between the MH and MUH categories (*p* < 0.05) (Table 1).

### 3.2. Dietary Intake, DII, and DIL

As shown in Table 2, there was no significant mean difference in dietary intake between the MH and MUH categories, except for cholesterol. Cholesterol intake was significantly higher in the MUH than the MH group in the crude model; however, after controlling energy intake, there was no significant mean difference (*p* > 0.05). Moreover, DII and DIL scores were insignificant in the crude or adjusted model in the MUH and MH groups (*p* > 0.05). Furthermore, other micronutrients and macronutrients were insignificant in the crude model or after adjustment with confounders (Table 2). The HOMA-IR index was significantly higher in the MUH than the MH group in the crude and adjusted model. HDL was significantly higher in the MH than the MUH group in the crude and adjusted model.

### 3.3. Association of TGF-*α* and IL-1*α* and MH and MUO Phenotypes

The association of TGF-*α* and IL-1*α* with MUH compared to MH, analyzed using the binary logistic regression model analysis in crude and adjusted models, are presented in Table 3. In the crude model, an association was observed between the MUH group and IL-1*α*, where the MUH group had 2.884-fold increased odds of IL-1*α* compared to the MH group (OR = 2.884, 95% CI = 0.957, 8.691, and *p* = 0.060). Additionally, in model 1, after adjustment with confounders (age, physical activity, BMI, and energy intake), the MUH group had a 3.441-fold increased odds of IL-1*α* compared to the MH group (OR = 3.441, 95% CI = 0.947, 12.502, and *p* = 0.060). In model 2, after further adjustment with confounders (FM and FFM), the MUH group had 3.566-fold increased odds of IL-1*α* compared to the MH group (OR = 3.566, 95% CI = 0.947, 13.430, and *p* = 0.060); however, all of the models mentioned above were not significant.

For TGF-*α*, in the crude model, the MUH group had a 1.169-fold increased odds of TGF-*α* compared to the MH group (OR = 1.169, 95%CI = 0.561, 2.439, and *p* = 0.677); while in model 1, after adjustment with confounders (age, physical activity, BMI, and energy intake), the MUH group had 1.216-fold increased odds of TGF-*α* compared to the MH group (OR = 1.216, 95% CI = 0.567, 2.608, and *p* = 0.615). In model 2, after further adjustment with confounders (FM and FFM), the MUH group had 1.245-fold increased odds of TGF-*α* compared to the MH group (OR = 1.245, 95%,CI = 1.074, 2.702, and *p* = 0.079); although all the aforementioned models were not significant.

### 3.4. Associations of DII and DIL with MH and MUO Phenotypes

The associations of DII and DIL with MH and MUH, analyzed using binary logistic regression analysis, are presented in Table 4. In model 1, after adjusting for confounders (age, physical activity, BMI, and energy intake), a significant association was observed between the MH group and DII, where the MH group had a 2.142-fold increased odds of DII compared to the MUH group (OR = 2.142, 95%CI = 1.421, 2.850, and *p* = 0.040).

For DIL in model 1, an association was observed between MH and DIL intake, where the MH group had a 2.674-fold increased odds of DIL compared to the MUH group, but this was not significant (OR = 2.674, 95%CI = 1.001, 3.471, *p* = 0.055) (Table 4).

### 3.5. Associations of TGF-*α* and IL-1*α* with DII, DIL, MH, and MUO Phenotypes

The association between DIL, DIL, MH, and MUH phenotypes with TGF-*α* and IL-1*α* is shown in Table 5. The positive odds remained between the MH group and DII, and IL-1*α* made the *p* value nonsignificant (OR = 0.946, 95% CI = 0.886, 1.000, and *p* = 0.072). Also, the positive odds were remained between MH and DIL intake, which IL-1*α* made the *p* value nonsignificant (OR = 1.00, 95% CI = 1.000, 1.000, and *p* = 0.221) (Table 5). In the association between MH and DIL, IL-1*α* may play a role in DIL intake. It seems that TGF-*β* does not have a role with these variables due to the significant *p*-value.

### 3.6. Correlations between DII and DIL with TGF-*α* and IL1

**Figure 1:**
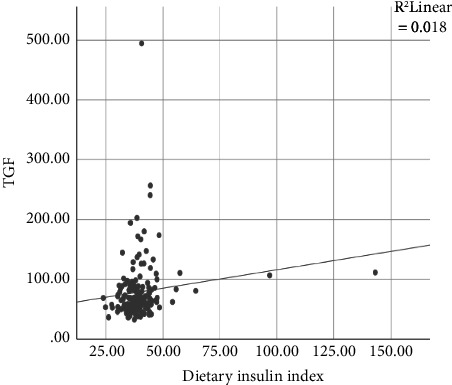
Nonsignificant Pearson correlation between TGF-*α* and dietary insulin index; *p* value: 0.06; r: 0.136.

According to Figure 1, nonsignificant positive correlation was observed between DII with TGF-*α* (*r* = 0.136, *p* = 0.060).

## 4. Discussion

In this study, we investigated the effect of inflammatory mediators on the relationship between dietary insulin indices and the metabolic phenotypes of overweight and obese women. Body mass, BMI, FMI, BF (%), BFM, trunk fat, WC, WHR, and WHtR were lower in the MH group. Moreover, the MH group had increased odds of DII which IL-1*α* may play a role. There was no association between MUH and inflammatory factors. To our knowledge, the present study is the first study to investigate the association of inflammatory factors, IL-1*α* and TGF-*α*, with DII and DIL in MUH/MH phenotypes.

There was no difference in DII and DIL between the MUH and MH groups in the present study. High DII facilitates the progression of obesity because it reduces fat oxidation and increases carbohydrate oxidation by increasing insulin secretion, which ultimately increases fat storage [45]. The mean DII in our study was 39.662 in the MH group and 39.534 in the MUH group. Also, the average DIL in the MH group was 101182.011 and MUH was 104795.485, while there was no significant difference between the two groups in terms of DII and DIL. In a previous study by Nimptsch et al., the median of DII was 42.8 and 677 for DIL for women [35]. The level of DIL in our study was higher than in other studies [46]. Accordingly, this difference might be due to different methods of calculating the DIL and/or the high intake of carbohydrate-containing foods. According to the literature, the amount of carbohydrate consumption in the Iranian diet is higher than in other nations; in fact, Iranians consume about 62% of their energy from carbohydrate-containing foods [32].

It has been demonstrated that inflammation plays an important role in developing obesity. Indeed, when energy intake is high and there is a positive energy balance, fat is stored in visceral adipose tissue. Since the ability of these cells to store fat is limited, these cells eventually become hypertrophic and disrupted [47]. Dysfunctional adipose tissue cells secrete various cytokines, including IL-1*α* and ΤΝF-*α*, as well as chemokines such as *Monocyte chemoattractant protein*-*1* (MCP-1) [48]. Various local or systemic cytokines are increased in obesity, including IL-1*α* [49, 50], TNF-*α* [51], leptin [52], and IL-6 [12, 53], while IL-1*α*, in combination with TNF-*α* and IL-6, is involved in the pathology of atherosclerosis in obese individuals. These cytokines can lead to low-grade systemic inflammation [54], in addition to causing IR in cells through inflammatory pathways inside the cell (such as NF-k*β*) [54, 55]. Esser et al. [56]. showed that secretion and expression of IL-1*α* were higher in MUO than in MHO [56].

Significant associations were observed between the MH group and DII, and it appears that IL-1*α* may have a role in this association. Amiri et al. observed that serum IL-1*α* concentrations may be due to abdominal obesity and not related to the presence or absence of metabolic health [57]. Therefore, this evidence suggests that a high DII intake can lead to obesity in women [32], and the increase in abdominal adipose tissue may alter serum IL-1*α* concentrations irrespective of the presence or absence of a healthy metabolic profile [57]. In addition, it has been previously found that IL-*α* reduced insulin secretion from the pancreatic cells of rats [58]. Other *in vitro* studies have shown that exposure to IL-1*α*, depending on its concentration and duration, can potentiate [59] and inhibit insulin secretion [60].

Consequently, it is viable that lower concentrations of IL-1*α* in MH compared to MUH individuals may have a positive effect on insulin and consequently on metabolic health. This may explain, at least partially, the association of IL-1*α* with the relationship between MH and DII. In this study, the HDL concentration in the MH group was higher than the MUH group, and BMI and HOMA-IR were lower in the MH group, but in contrast to other studies, there was no significant difference in TG and FBS between the two groups [36]. The mean HDL concentration in the MH group was 53.108 and in the MUH group was 45.043, which is lower than normal HDL, and the difference in HDL in the two groups was significant (normal range in women: above 50 mg/dL). IR was significant in both groups, and in the MUH group, IR levels were higher than in the MH group.

On the other hand, their HDL concentration was lower than normal in the MUH group. According to a previous study, an insulinogenic diet increases insulin secretion as a function of underlying IR [35]. Thus, it may be posited that this lower HDL in the MUH group (which has a higher IR status than the MH and DII = 39,534) may be due to adherence to the high DII intake [31]. Indeed, Nimptsch et al. [35] reported a significant positive association between DIL/DII and plasma TG and HDL concentrations in obese people. In contrast, Anjom-Shoae et al. [32] reported that higher DII was associated with lower HDL and higher TG concentrations (positive association).

In the studies we reviewed, we found that TGF-*α* did not increase in obese individuals, while, on the other hand, these inflammatory markers are higher in lean individuals [61]; as in our study, no association was found between TGF-*α* and the MUH phenotype.

The main finding of the current study is that an MH phenotype in overweight and obese women is significantly associated with DII, and IL-1*β* may play a role in this association. The strengths of this study were the use of the validated FFQ questionnaire and that the study was in women only, thereby allowing more nuanced insight into the effects of inflammatory markers, IL-1*α* and TGF-*α*, and their association with dietary insulin load and dietary insulin index. However, despite the strengths of this study, one of its limitations is the large age range of participants. Thus, we advocate that further research utilize participants with a smaller age range. Moreover, examining other factors such as menopause and the hormonal status of participants could affect the accuracy of the results and must be considered in subsequent investigations. Although our investigation included the total number of participants established in our sample size calculation, it could be argued that the smaller number of participants in the MH group may have affected the power of the analysis. Finally, the most important limitation of this study is its cross-sectional design, which precludes causal inferences from being made; thus, prospective studies are needed to confirm our findings.

## 5. Conclusion

In summary, an MH phenotype in overweight and obese women is associated with DII and DIL intake, and IL-1*β* might play a role in this association. However, no relationships were found between MUH phenotype and inflammatory factors.

## Figures and Tables

**Table 1:** Demographic characteristics of MH and MUH overweight and obese women (*N* = 228).

Variables	MH (*n* = 63)	MUH (*n* = 165)	*P* value*∗*	*P* value‡*∗∗*
	(Mean ± SD)			
Quantitative variables demography^a^				

Demographic characteristic				
Age (years)	36.392 ± 1.107	36.139 ± 0.662	0.763	0.847
PA (MET)	949.773 ± 297.378	1327.518 ± 180.230	0.260	0.289

Anthropometry and body composition				
Height (cm)	161.1825 ± 5.20879	161.3079 ± 5.78749	0.881	0.881
Body mass (kg)	75.022 ± 1.520	81.112 ± 0.926	**0.001**	**0.001**
BMI (kg/ **m**^2^)	28.678 ± 0.517	31.064 ± 0.315	**<0.001**	**<0.001**
FMI	11.6435 ± 2.54098	13.0804 ± 2.74453	**<0.001**	**<0.001**
BF (%)	39.120 ± 0.870	41.341 ± 0.530	**0.032**	**0.032**
BFM (Kg)	29.593 ± 1.004	33.932 ± 0.612	**<0.001**	**<0.001**
Trunk fat (kg)	14.639 ± 0.498	16.866 ± 0.303	**<0.001**	**<0.001**
WC (cm)	89.263 ± 2.511	97.241 ± 1.530	**0.008**	**0.008**
HC (cm)	110.953 ± 1.129	113.437 ± 0.687	**<0.001**	0.064
WHR	0.914 ± 0.008	0.944 ± 0.005	**0.002**	**0.002**
WHtR	0.580 ± 0.008	0.620 ± 0.005	**<0.001**	**<0.001**
HDL (mg/dL)	53.108 ± 2.410	45.043 ± 2.211	**0.029**	**0.029**
LDL (mg/dL)	87.484 ± 5.840	97.446 ± 5.358	0.255	0.255
HOMA_IR index	2.545 ± 0.255	3.526 ± 0.234	**0.013**	**0.013**

Inflammatory parameter				
TGF (ng/ml)	77.826 ± 10.195	70.715 ± 9.352	0.639	0.639
IL-1*α* (ng/ml)	2.530 ± 0.205	3.000 ± 0.188	0.128	0.128

Qualitative variables^b^ᵇ				
Marital status				
Single (%)	16 (32.0)	34 (68.0)	0.425	0.533
Married (%)	46 (26.3)	129 (73.7)		

Supplement				
Yes	27 (26.5)	75 (73.5)	0.239	0.193
No	26 (34.7)	49 (65.3)		

Education				
Illiterate (%)	1 (33.3)	2 (66.7)	0.510	0.472
Under diploma (%)	4 (15.4)	22 (84.6)		
Diploma (%)	24 (27.9)	62 (72.1)		
Bachelor and above (%)	33 (30.0)	77 (70.0)		

Economic status				
Low class (%)	15 (27.8)	39 (72.2)	0.377	0.448
Middle class (%)	31 (32.0)	66 (68.0)		
High class (%)	13 (21.7)	47 (78.3)		

MH: metabolically healthy, MUH: metabolically unhealthy, PA: physical activity, SBP: systolic blood pressure, DBP: diastolic blood pressure, BMI: body mass index, FFM: fat-free mass, FFMI: fat-free mass index, FMI: fat mass index, BF: (%) body fat percentage, BFM” body fat mass, WC: waist circumference, HC: hip circumference, WHR: waist-to-hip ratio, WHtR: waist-to-height ratio, FBS: fasting blood sugar, Chol: cholesterol, TG: triglyceride, HDL: high-density lipoprotein, LDL: low-density lipoprotein, GOT glutamic oxaloacetic transaminase, GPT glutamate pyruvate transaminase, HOMA: homeostatic model assessment, TGF: transforming growth factor-beta, IL-1*α*: interlukin1, and SD: standard deviation. Data are presented as mean ± standard deviation (SD) or percent. ^‡^Collinear variables did not enter into the model, and this *P* value was obtained from ANCOVA analysis. *∗P* value was obtained from independent *T*-test. *∗∗P* value was obtained from ANCOVA test, variable adjust for age, physical activity, energy intake, and BMI. Chi-square and analysis of variance were used for qualitative and quantitative variables, respectively, and the *P* value was set to <0.05. ^a^mean ± SD. ^b^ᵇ*n* (%).

**Table 2:** Dietary intakes, dietary insulin index, and dietary insulin load among MH/MUH phenotype category in overweight and obese women (*N* = 228).

Variables	MH (*n* = 63	MUH (*n* = 165	*∗P* value	*∗∗P* value
	(Mean ± SD)			
Insulin components				
Dietary insulin index	39.662 ± 1.192 39.534 ± 0.751	0.927	0.974	
Dietary insulin load	101182.011 ± 5133.94 104795.485 ± 3236.219	0.552	0.515	

Macronutrients and total energy intake				
Energy (kcal)	2530.600 ± 759.357 2652.461 ± 761.313	0.287	-	
Cho (% energy)^a^	58.129 ± 0.898 56.039 ± 0.565	0.059	0.051	

Micronutrients				
Chol (mg/d)	234.910 ± 10.911 257.710 ± 6.867	**0.032**	0.079	
HOMA-IR index	2.545 ± 0.255 3.526 ± 0.234	**0.013**	**0.013**	
HDL	53.108 ± 2.410 45.043 ± 2.211	**0.029**	**0.029**	

MH: metabolically healthy, MUH: metabolically unhealthy, Cho: carbohydrate, Chol: cholesterol, and SD: standard deviation. Data are presented as mean ± standard deviation (SD) or percent. *∗P* value was obtained from independent *T*-test. *∗∗P* value was obtained from ANCOVA test, variable adjust for total energy, and the *P* value was set to <0.05. ^a^Energy percent (%).

**Table 3:** Association of TGF-*α*, IL-1*α*, metabolically healthy, and metabolically unhealthy obesity among overweight and obese women (*N* = 228).

MUH		OR	95% CI	*P* value
Crude model	IL-1*α*	2.884	0.957^b^ᵇ, 8.691^a^	0.060
Model 1		3.441	0.947, 12.502	0.060
Model 2		3.566	0.947, 13.430	0.060
Crude model	TGF-*α*	1.169	0.561, 2.439	0.677
Model 1		1.216	0.567, 2.608	0.615
Model 2		1.245	1.074, 2.702	0.079

MUH metabolically unhealthy, OR odds ratio, and CI confidence interval. *P* values are reported based on the binary logistic regression test and are considered significant at <0.05. For TGF-*α*, lower amounts of the median (TGF-*α* <66.25) are considered as the reference group. For IL-1*α*, lower amounts of the median (IL-1*α* <2.75) are considered as the reference group. For metabolically healthy and metabolically unhealthy (outcomes), MH (metabolically healthy) is considered the reference group. Model 1: adjusted for age, physical activity, BMI, and energy intake (Kcal). Model 2: further adjustment with FM and FFM. ^a^Upper. ^b^ᵇLower.

**Table 4:** Association of DII, DII and metabolically healthy, and metabolically unhealthy Obesity in overweight and obese women (*N* = 228).

MH		OR	95% CI	*P* value
Crude model	DII	1.675	0.862^b^, 3.255^a^	0.128
Model 1		2.142	1.421, 2.850	**0.040**
Crude model	DIL	0.763	0.396, 1.470	0.419
Model 1		2.674	1.001, 3.471	0.055

MH metabolically healthy, OR odds ratio, CI confidence interval, DII dietary insulin index, and DIL dietary insulin load. P values are reported based on the binary logistic regression test and are considered significant at <0.05. For the dietary insulin index, upper amounts of the median (DII> 38.8228) are considered the reference group. For dietary insulin load, upper amounts of the median (DIL> 97155.6995) are considered the reference group. For metabolically healthy and metabolically unhealthy (outcomes), MUH (metabolically unhealthy) is considered the reference group. Model 1: adjusted for age, physical activity, BMI, and energy intake (Kcal). ^a^Upper. ^b^ᵇLower.

**Table 5:** The association of TGF-*α* and IL-1*α* with DII and DIL, and metabolically healthy with metabolically unhealthy as the reference group in overweight and obese women (*N* = 228).

		Inflammatory marker	OR	95% CI	*P* value
MH	DII	TGF-*α*	5.915	1.147^b^, 24.497^**a**^	**0.021**
		IL-1*α*	0.946	0.886, 1.000	0.072
MH	DIL	TGF-*α*	12.597	1.043, 17.21	**0.032**
		IL-1*α*	1.000	1.000, 1.000	0.221

MH metabolically healthy, OR odds ratio, CI confidence interval, DII dietary insulin index, and DIL dietary insulin load. *P* values are reported based on the binary logistic regression test and are considered significant at <0.05. For the dietary insulin index, upper amounts of the median (DII> 38.8228) are considered the reference group. For dietary insulin load, upper amounts of the median (DIL> 97155.6995) are considered the reference group. For metabolically healthy and metabolically unhealthy (outcomes), MUH (metabolically unhealthy) is considered the reference group. TGF-*α* adjusted for age, physical activity, BMI, energy intake (Kcal), and IL-1*α*. ^a^Upper. ^b^Lower.

## Data Availability

The data that confirm the findings of this study are available from Khadijeh Mirzaei, but restrictions apply to the availability of these data, which were used under license for the current study, and so are not publicly available. Data are available from the authors upon reasonable request and with permission of Khadijeh Mirzaei.
